# A Type of Annulus-Based Energy Balanced Data Collection Method in Wireless Rechargeable Sensor Networks

**DOI:** 10.3390/s18093150

**Published:** 2018-09-18

**Authors:** Chao Sha, Qin Liu, Si-Yi Song, Ru-Chuan Wang

**Affiliations:** 1 School of Computer Science, Software and Cyberspace Security, Nanjing University of Posts and Telecommunications, Nanjing 210003, Jiangsu, China; liuqinyz@163.com (Q.L.); wangrc@njupt.edu.cn (R.C.W.); 2 School of Oversea Education, Nanjing University of Posts and Telecommunications, Nanjing 210003, Jiangsu, China; ssong04@nyit.edu

**Keywords:** wireless sensor networks, annulus-sector, energy balance, data forwarding strategy, wireless charging vehicles

## Abstract

With the increasing number of ubiquitous terminals and the continuous expansion of network scale, the problem of unbalanced energy consumption in sensor networks has become increasingly prominent in recent years. However, a node scheduling strategy or an energy consumption optimization algorithm may be not enough to meet the requirements of large-scale application. To address this problem a type of Annulus-based Energy Balanced Data Collection (AEBDC) method is proposed in this paper. The circular network is divided into several annular sectors of different sizes. Nodes in the same annulus-sector form a cluster. Based on this model, a multi-hop data forwarding strategy with the help of the candidate cluster headers is proposed to balance energy consumption during transmission and to avoid buffer overflow. Meanwhile, in each annulus, there is a Wireless Charging Vehicle (WCV) that is responsible for periodically recharging the cluster headers as well as the candidate cluster headers. By minimizing the recharging cost, the energy efficiency is enhanced. Simulation results show that AEBDC can not only alleviate the “energy hole problem” in sensor networks, but also effectively prolong the network lifetime.

## 1. Introduction

In the era of big data and artificial intelligence, wireless sensor networks (WSNs) with sensing, computing and communication ability have gradually become more and more important. In general, nodes send their data to a sink which is located at the center of the network via single-hop or multi-hop transmission. However, the energy of nodes are limited and nodes nearer to the sink have more data forwarding tasks than other nodes, thus, the energy of some nodes is exhausted quickly and the energy holes inevitably appear [1,2]. After an energy-hole appears no more data can be delivered to the sink [3,4]. In addition, nodes near the energy-hole are required to bear the data load of those dead nodes so that their energy consumption level will increase more rapidly [5]. As a result, the network becomes disconnected and the network lifetime ends prematurely although vast amounts of residual energy may remain unused.

To solve the energy hole problem, most of the existing works focus on the optimization of the network architecture. To improve the energy efficiency and enhance the stability of the network, clustering has been applied in most multi-hop networks [6]. There have been many unequal clustering approaches proposed by researchers. For example in [7], Chen et al. proposed an unequal cluster-based routing protocol to mitigate the hot-spot problem. The cluster heads (CHs) forward data to a sink with the help of a greedy geographic and energy-aware routing protocol. However, this may aggravate the burden of some CHs. Moreover, the data is aggregated into fixed-length packets which is not suitable for networks with massive sensing data. Thus, how to further optimize the deployment of nodes as well as the data forwarding strategy are important for mitigating the energy hole problem and improving energy efficiency.

Many studies have shown that energy harvesting from natural sources, such as wind, solar, vibration and thermal can effectively improve network performance and prolong network lifetimes. However, the effect of energy harvesting mainly depends on the environment. For example, in a solar harvesting system, the amount of harvested energy is determined by the duration and strength of the solar radiation. Thus, energy harvesting from the environment is not entirely practical for WSNs [8]. On the other hand, Kurs et al. have demonstrated that by utilizing magnetic resonant coupling technology, wireless power transfer is both feasible and practical [9]. Nowadays, with the rapid development of energy harvesting and wireless recharging technologies, the advantages of Wireless Rechargeable Sensor Networks (WRSNs) are becoming more and more obvious in extending WSNs’ lifetime and improving system robustness [10,11,12]. In a realistic scenario, a Wireless Charging Vehicle (WCV) travels across the network and charges all the rechargeable sensor nodes [13]. The recharging sequences are often calculated in advance so that nodes can be recharged before energy depletion [14]. Each node would be charged only once by a WCV in one charging cycle [13]. After visiting all the nodes, the WCV returns to the service station to recharge. Ideally, the lifetime of a WRSN can thus be extended infinitely for perpetual operation [14]. A proof-of-concept WRSN prototype has been established by Peng et al. [15], and experiments have been conducted to evaluate its feasibility as well as the energy replenishment performance in a small-scale WSN. However, the main drawback of energy harvesting is the low recharging efficiency, since the power output of energy harvesting devices is relatively low compared to the energy consumption for sensing and communications. Some serious problems will appear if these limitations are not addressed. For example, some nodes will die before the arrival of the Wireless Charging Vehicle (WCV), or the recharging burden of the WCV is so heavy that there is not enough power for it to return to the service station [14].

In this paper, we investigate two typical energy balance algorithms and analyze their advantages and disadvantages. Liu et al. [16] proposed an Energy-Balancing unequal Clustering Approach for Gradient based routing (EBCAG). The nodes are dynamically organized into clusters, which achieves a balance between CHs and member nodes in a cluster. However, this method increases the energy cost of the whole network. In [17], the authors designed an Energy Balancing Cluster Head (EBCH) method to balance the energy consumption of CHs in different annuli. However, they didn’t consider the unbalanced workload or the energy consumption between CHs and common nodes. To solve the above problems, a type of annulus-based energy balanced data collection method is proposed in this paper. Without loss of generality, the circular network is firstly divided into several annulus-shaped sectors of different sizes. Nodes in the same annulus-sector form a cluster. The main contributions of this paper are the following:

- We analyse the energy consumption of nodes in each annulus and conclude that the CHs in different annuli should be selected from different sizes of regions, which can balance their energy consumption. According to this conclusion, the length of the radius of each Region for Candidate Cluster Headers (RCCH) is calculated.
- A data forwarding strategy with the help of the Candidate Cluster Headers (CCHs) is proposed to reduce the possibility of buffer overflow during data uploading. With the collaboration of CCHs, the energy consumption of CHs is reduced and the energy consumption between CHs and CCHs is approximately balanced in a round of data gathering.
- In order to achieve balanced energy consumption between CHs and common nodes, we adopt wireless charging technology. In each annulus, there is a WCV that is responsible for recharging the CHs and CCHs in the same annulus under the constraint of minimizing the recharging cost. Therefore, the energy efficiency of the network is improved and the energy hole problem is effectively alleviated.

It is worth mentioning that the proposed method is not only adaptable for circular networks, but also can be applied to networks of other shapes. For example, in a large-scale farmland with regular or irregular shapes, the static sensor nodes are randomly deployed and form several clusters. They upload data to the base station (the monitoring center) in a multi-hop way. In order to maintain the long-term running of the network, a number of WCVs move in this farmland according to some predetermined trajectories (e.g., the field ridges) and recharge the relay nodes and cluster heads. Thus, the growth of crops can be monitored for a long time.

The remainder of this paper is organized as follows: related works and the network model are described in Section 2 and Section 3, respectively. In Section 4, we propose a type of data forwarding strategy with the help of the candidate cluster heads. Then, a multi-MCV based recharging scheme for maximal benefit is described in Section 5 and experimental results are shown in the next section.

## 2. Related Works

To alleviate the “energy hole problem”, many studies have been done for achieving energy balance and maximizing the network lifetime. In this section, we introduce some typical studies on clustering, mobile sink-based data collection and wireless recharging for nodes in WSNs. 

### 2.1. Clustering Optimization Algorithms

Low Energy Adaptive Clustering Hierarchy (LEACH) was the first clustering protocol for WSNs that achieved low energy dissipation and latency without sacrificing application-specific quality [18]. Each node in the cluster sends its data to a local cluster-head, and the periodic CH selection strategy evenly balances the energy consumption among all the nodes to some extent. However, the size of clusters in LEACH are equal to each other, and each CH transmits data to the sink via a single hop. This causes more energy consumption for communication. For this reason, the uneven clustering model was then proposed in [19] for balancing the energy consumption of CHs. However, the disadvantage of this model is that it can only be applied in WSNs with two-hop inter-cluster communication.

**Figure 1:**
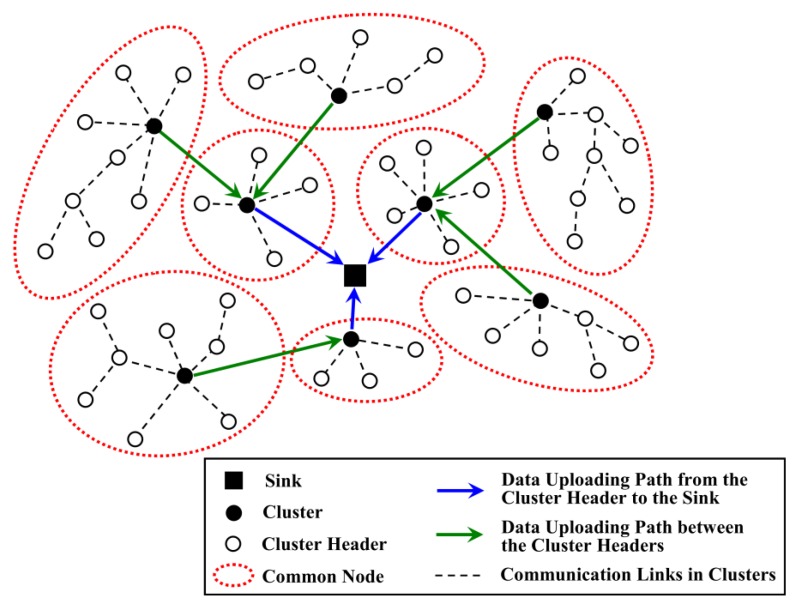
A type of uneven clustering network model.

In [20], the size of clusters located around the sink is smaller than those far away from the sink, as shown in Figure 1. In this case, the cluster heads near the sink consume less energy while collecting data from their direct children, so they have enough energy to forward data uploaded from other clusters. Although this type of network structure efficiently balances the energy consumption of the whole network and alleviates the energy hole problem near the sink, the workload between the CHs and common nodes is still unbalanced. Moreover, the unsuitable location of the cluster header may also increase the energy consumption for communication between this CH and its members. Liu et al. regarded that inter-cluster energy consumption is more than intra-cluster energy consumption in a large-scale WSN [16]. Therefore, they designed EBCAG to achieve energy balance among CHs and to decrease the total energy consumption of nodes. Each node has a gradient value which determines the optimal radius of the cluster as well as the routing strategy for CHs. Nevertheless, the energy consumption for calculating the optimal radius is still higher, which decreases the energy efficiency of the whole network.

To solve the unbalanced energy consumption problem in a randomly distributed network, a self-organized clustering method was proposed in [21]. The cluster radius is calculated according to the density of nodes as well as the average distance between each node and the base station. It effectively balances the energy consumption among clusters with different density. However, each node must maintain a cluster information table, which increases the computational complexity. Moreover, due to the unreasonable local optimal routing strategy, the load difference between cluster heads is still large.

In [22], the network is divided into unequal clusters and there are three types of nodes in each cluster. The data collected by the ordinary nodes are no longer sent to the cluster header directly but rather forwarded by the leader nodes to the CH. This effectively saves the intra-cluster energy and prolongs the network lifetime, but the CH selection strategy of this algorithm is a little complicated. Gupta et al. employed a non-uniform clustering algorithm to mitigate the energy hole problem. In addition to the hop distance as well as the residual energy, the number of neighbor nodes is also considered during the CH selection process, which prolongs the network lifetime [23]. Moreover, to further save node energy, the CH selection is not executed frequently and a sleep scheduling mechanism is also adopted in clustering.

For circular networks, Lakshminarayanan et al. proposed the EBCH method to avoid the energy hole problem [17]. The network is divided into several coronas, and then different sizes of clusters are formed in each sector. An optimum load distribution model is designed in which the CHs send some data to the sink directly and the rest of the data is forwarded to the CHs of the inner coronas to balance the energy consumption among CHs. Unfortunately, this model is not suitable for a large-scale network. The network model proposed in this paper is similar to that in EBCH. Different from the mixed routing approach, in this paper, the CH send its data to the candidate cluster headers (RCHs) in the adjacent inner annulus. This not only improves the network throughput, but also balances the energy consumption of the CHs and the RCHs. Moreover, our algorithm can be employed in large-scale networks.

### 2.2. Mobile Sink Based Data Collection Methods

With the help of one or more mobile sinks, data can be uploaded to the base station within one hop. This greatly reduces the energy consumption during transmission, and the “energy hole problem” can also be mitigated to some extent. Furthermore, the hotspots around the sink change as it moves, and the workload around the sink can also be distributed to more nodes, which balances the energy consumption [24].

**Figure 2:**
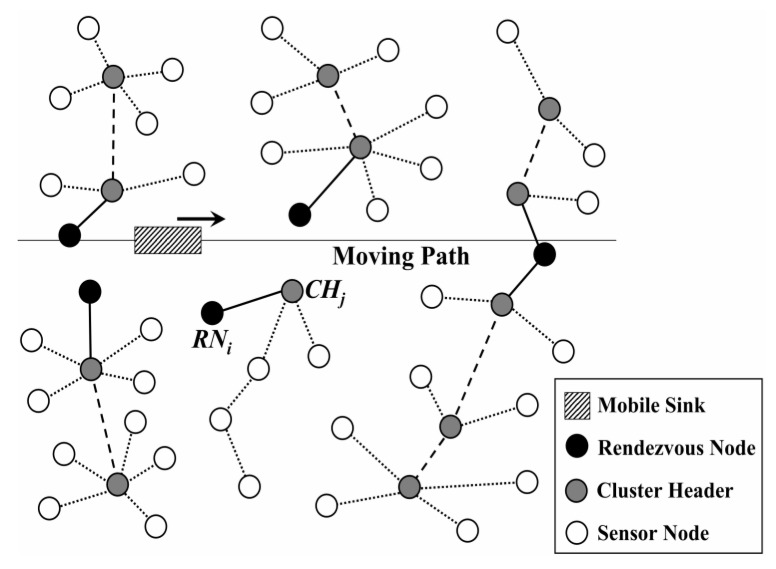
Network structure of MobiCluster [27].

To reduce energy consumption during data collection, Gao et al. [25] have divided the sensor nodes into sub-sink nodes which are in a direct communication area or far-away nodes that are within the distance of the multi-hop communication area. Sinks move along a fixed path to gather as much data as possible. Similar to this, a type of Data Gathering method based on one mobile sink moving along fixed traverse points (DGFP) has been proposed in our previous work [26]. With the help of the sensing and coverage models, an optimal trajectory for the mobile sink was built to achieve a balanced energy consumption. In addition, a sleep scheduling strategy has also been introduced to further reduce energy consumption. Moreover, Charalampos et al. [27] proposed a type of rendezvous-based data collection method, named MobiCluster. The mobile sink is mounted on public buses circulating within urban environments on fixed trajectories and near-periodic schedules. Nodes are often deployed in urban areas in proximity to public transportation vehicle routes. To balance energy consumption, nodes located near the sink trajectory are grouped in small-sized clusters while nodes located farther away are grouped in clusters of larger size, as shown in Figure 2. In this case, the overhead of multi-hop data relaying to the edge rendezvous nodes is minimized [27]. However, the trajectory of the mobile sink is not controllable and its route does not adapt to specific WSN deployments.

In [28], the network is uniformly divided into sectors, and the nodes in each sector form a cluster. All the CHs link into a chain and the CH nearest to mobile sink is selected as the leader. It forwards data sent from other CHs to the mobile sink. This strategy mitigates the hotspots problem to some extent, and the CHs far away from the sink consume less energy for data transmission. However, the packet delay is increased due to a large number of hops between the nodes and the sink. In addition, data will be dropped if one node in the chain fails. In [29], a Mobile Sink-based adaptive Immune Energy-Efficient clustering Protocol (MSIEEP) was proposed to alleviate the energy hole problem. In this algorithm, an adaptive immune algorithm was used to find the sojourn locations of the mobile sink as well as the optimum number of CHs. Thus, the total energy dissipated in communication and the overhead control packets of all sensor nodes can be minimized. Although the lifetime of network is improved, the unbalanced load problem still exists. With the help of multiple mobile Sinks, Gharaei et al. proposed a two-stage greedy algorithm to obtain the upper bound of an optimal cluster size interval [30]. Then, by designing the circular motion of the mobile sinks with varied velocity, the energy consumption of CHs in different coronas achieves balance. However, the optimal sojourn time for the mobile sinks to stay at different locations was not discussed in this paper.

On the other hand, one should not ignore the energy consumption when frequently broadcasting the location of the mobile sink. To solve this problem, several mobile relay nodes were introduced in the network and they were regarded as the mobile sinks of the sub-networks [31]. These mobile relay nodes independently collect data in each sub-network and forward them to the static sink. However, due to their limited energy, node failure may occur frequently, which causes the disconnection of the whole network. Moreover, Gu et al. have also divided the network into multiple groups based on nodes’ locations and data generation rates [32]. In each group, the mobile data collector visited each node at a fixed frequency to avoid buffer overflow.

### 2.3. Wireless Recharging Strategies

As mentioned above, due to the breakthrough in energy transfer technology, there have been many studies that employ mobile Wireless Charging Vehicles (WCVs) to prolong the lifetime of WSNs [33,34,35]. In [33], the authors envisioned employing a mobile vehicle to periodically visit each node and charge it wirelessly. By always keeping the energy level of each node above the minimum threshold, the network can run permanently. The objective of this method is to maximize the ratio of the time for the WCV on staying at the service station to the time of the recharging cycle, and the authors proposed a near-optimal solution. Zhang et al. proposed an optimal data gathering method for the cluster-based network by introducing the Energy-Harvesting nodes (EHs) [34]. The idea of this algorithm is to find the relationship between the best location of CHs and the best location of EHs. However, it assumes that the EHs can harvest energy from the environment and the location of them are adjustable. This is unrealistic in real applications.

Wang et al. considered the movement costs and capacity constraints of the WCV [14]. In their method, the sensor nodes are organized into clusters and one data gathering vehicle as well as multiple charging vehicles are introduced into the network. To achieve the aim of balancing energy consumption and reducing data collection latency, they formulated recharge optimization into a Profitable Traveling Salesmen Problem with the constraints of battery capacity and designed a simple Greedy Algorithm (GA) as well as a three-step Adaptive Algorithm (AA) to solve this problem. In [35], the author employed some Mobile Data Collectors (MDCs) to visit each nodes and collect their data. Different from other mobile Sinks based data gathering strategies, these MDCs can also recharge nodes. To minimize the energy consumption and satisfy the packet-delay constraint, they try to find out the optimal path of these MDCs, so a two-phase path selection algorithm was proposed. 

Considering the cost of data collection as well as the computing overhead for routing, we combine the static data collection method with the wireless mobile charging strategy. In each annulus, there is a WCV that is only responsible for recharging the cluster headers or the candidate cluster headers. In addition, with the help of the candidate cluster headers, the sensed data is uploaded to the static sink through a few hops in a distributed manner. By balancing the energy consumption between the common nodes and CCHs, the network lifetime can be effectively prolonged. 

## 3. Network Model

**Figure 3:**
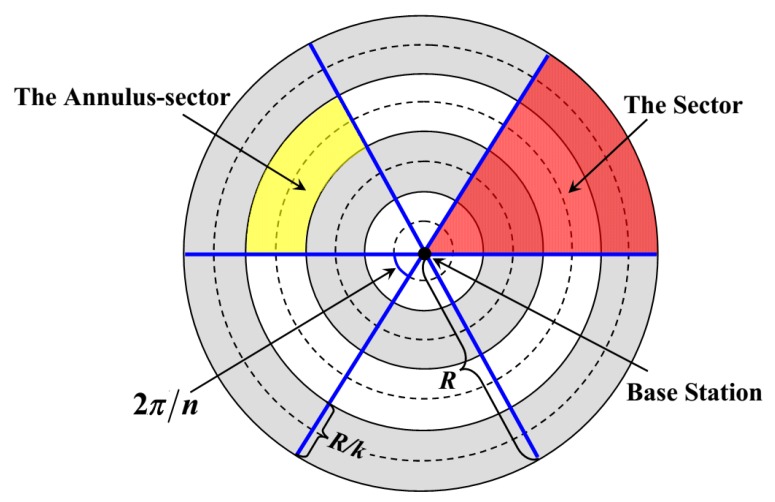
Network Model.

Similar to [17,30], the network is regarded as a circular region with a radius *R* and divided into *k* virtual concentric annuli with the same width. Meanwhile, this circular network is also divided into *n* sectors of the same size. That is to say, the network is composed of *nk* annular sectors. For example, to illustrate the model more clearly, we divide the network into four virtual concentric annuli, as shown in Figure 3. In addition, this network also consists of six sectors (the red area). Thus, there are 24 annulus-sectors (the yellow area) in the network. It is not difficult to know that, the number of these annulus-sectors will increase with the expansion of network scale, which enhances the flexibility of our model. 

A static base station (BS) is located at the center of the network, and *N* rechargeable sensor nodes are uniformly and randomly distributed in the network. Without loss of generality, the density of nodes is denoted as *ρ*. Moreover, the communication radius as well as the battery capacity of each node is defined as *r_t_* and *C*, respectively. It is regarded that nodes in each annulus-sector form a cluster, and they periodically send packets to the cluster heads (CHs) within one hop. Meanwhile, the CHs transmit all the collected data to BS in a multi-hop manner. What needs to be pointed out is that the CHs which are closest to the center of the network send their data directly to the BS. The definitions of parameters used in the network are given in Table 1.

It has been proved that the free space model cannot be completely applied to WSNs. Therefore, the classic energy consumption model for sensor nodes proposed by Heinzelman is adopted in this paper [18]. For simplicity, we ignore the energy consumption for computation and data storage.

(1) $$E_{t}=\left\{ \begin{array}{l} lE_{elec}+l\varepsilon_{fs}d^{2},d\leq d_{0}\\ lE_{elec}+l\varepsilon_{amp}d^{4},d>d_{0} \end{array}\right.$$

(2) $$E_{r}=lE_{elec}$$

*E_t_* and *E_r_* denote the energy consumption for sending and receiving *l* bit of data. In addition, *E_elec_* is the unit energy consumption of the transmitting and receiving circuit. *ε_fs_* and *ε_amp_* are regarded as the coefficients of energy consumption during communication in free space transmission and multi-path fading transmission, respectively. Moreover, *d* is defined as the Euclidean distance between the sender and the receiver, while *d*_0_ denotes the threshold distance.

According to the network model as well as the energy consumption formula, it is known that the CHs close to BS are heavily loaded nodes. They exhaust their energy quickly, and thus energy holes appear, so in this paper, the “Region for Candidate CHs” (RCCH) is firstly constructed in each annulus-sector which ensures the balance of energy consumption in CH election. Then, the data packets are forwarded by the collaborative Candidate Cluster Headers (CCHs), which further balance the load of CHs. Finally, *k* WCVs are assigned to *k* annuli, and the recharging strategy for the RCCH is proposed under the constraint of the maximal network lifetime. The energy consumption of the whole network is effectively balanced, and the “energy hole problem” is also alleviated to some extent. Without loss of generality, the moment when a dead node appears is defined as the end of the network lifetime.

## 4. Multi-Hop Data Forwarding Strategy Based on Annulus-Sectors

### 4.1. Cluster Header Selection

**Figure 4:**
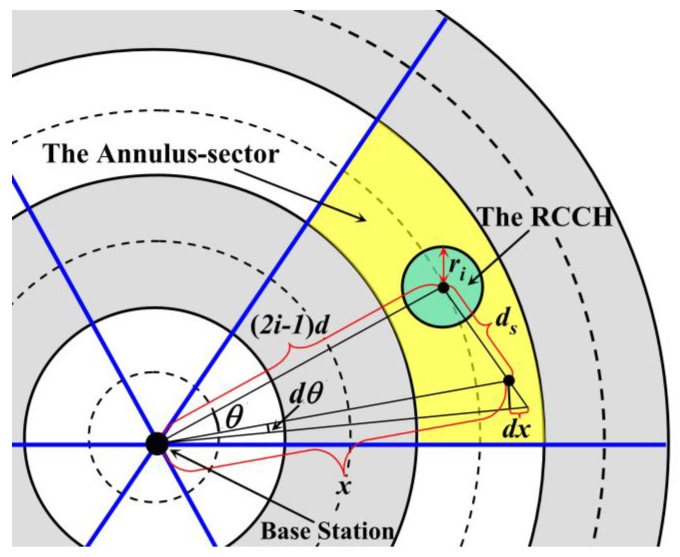
Analysis on energy consumption of the non-cluster heads.

According to [36], it is known that when the CH is located near the center of the cluster, it can effectively balance the energy consumption as well as prolong the lifetime of this cluster. Thus, the definition of the “Region for Candidate CHs” (RCCH) is firstly described. For the *j*-th annulus-sector in the *i*-th annulus, the intersection of its symmetry axis and the middle line of this area is regarded as the center of the RCCH (the green region in Figure 4, and the radius of this RCCH is denoted as *r_i_*. It is easy to know that *i* ∈ [1, *k*] and *j* ∈ [1, *n*]. All the cluster headers are selected from the nodes that are located in the RCCHs, and it is assumed that each node knows its own coordinates after network deployment. If the sensor node is located in the RCCH, it is regarded as the “Candidate Cluster Header (CCH)”, otherwise it is called a “Common Node (CN)”.

*W* is defined as the weight of each CCH, and the value of it is calculated by Equation (3). *α* and *β* are the adjustable parameters, and they meet *α + β* = 1. *e*_0_ and *e_r_* are defined as the initial and residual energy of the CCH respectively. Moreover, *d_tocenter_* is the Euclidean distance between the CCH and the center of the RCCH, and *δ* is an adjustable coefficient.

(3) $$W=\alpha \left(e^{r}/e_{0}\right)+\beta \left(\delta /d_{to\;center}\right)$$

At the beginning of each round of data collection, each CCH calculates its weight, and then the CCH with the maximal value of *W* in this RCCH is selected as the cluster head. Subsequently, the ID of this CH is broadcasted to all nodes in the same annulus-sector, and nodes that receive this message establish a one-hop communication link to the CH for uploading data. The definitions of data collection parameters are given in Table 2.

### 4.2. Energy Consumption of Nodes in an Annulus-Sector

#### 4.2.1. Energy Consumption of the Non-Cluster Heads

For the *j*-th annulus-sector of the *i*-th annulus, it is easy to know that the distance from the center of its RCCH to the base station (BS) is (2*i* − 1)*R*/2*k*, as shown in Figure 4. *x* and *d_s_* are defined as the Euclidean distances from any one node in this annulus-sector to BS or to the center of the RCCH, respectively. It is assumed that during a round of data collection, the amount of data collected by one node is *l* bit. Therefore, according to [37], the total energy consumption of the non-cluster heads in one annulus-sector (denoted as *E_ij_*) during a round of data gathering time is

(4) $$E_{ij}=2l\rho \int_{(i-1)R/k}^{iR/k}\int_{0}^{\pi /n}\left(E_{elec}+\varepsilon_{fs}d_{s}{}^{2}\right)xdxd\theta$$

So, in Figure 4, according to the law of cosines, it is known that:

(5) $$d_{s}{}^{2}=x^{2}+{\left(2i-1\right)}^{2}{\left(R/2k\right)}^{2}-2x\left(2i-1\right)\left(R/2k\right)\cos\theta$$

By combining Equations (4) and (5), we get:

(6) $$E_{ij}=4l\rho \varepsilon_{fs}{\left(\frac{R}{2k}\right)}^{4}\left(\frac{2\pi }{n}\left(i^{4}-{\left(i-1\right)}^{4}\right)+\frac{\pi }{n}{\left(2i-1\right)}^{3}-\frac{8}{3}\left(i^{3}-{\left(i-1\right)}^{3}\right)\left(2i-1\right)\sin\frac{\pi }{n}\right)+4l\left(2i-1\right){\left(\frac{R}{2k}\right)}^{2}\rho \left(\frac{\pi }{n}\right)E_{elec}.$$

#### 4.2.2. Energy Consumption of the Cluster Heads

The energy consumption of the CH is related to amount of date been transmitted as well as the distance between this CH and its next-hop forwarder. In addition, it is not difficult to know that nodes in the RCCH are all located near the center of the annulus-sector. Therefore, for two communicable CHs in adjacent annuli, it is regarded that the hop distance between them is approximately equal to the width of the annulus. For each CH, its energy consumptions when sending and receiving one bit of data are defined as *e_t_* and *e_r_*. Without loss of generality, their values are *E_elec_* + *ε_fs_*(*R*/*k*)^2^ and *E_elec_*, respectively.

The CH in the *k*-th annulus, only needs to receive data uploaded from the non-CHs in the same annulus-sector and forward them to the CH in the (*k* − 1)-th annulus. Therefore, during a round of data gathering, the energy consumption of this CH is:

(7) $$E_{CH}^{k}=l((N_{k}-1)e_{r}+N_{k}e_{t})$$

In Equation (7), *N_k_* is the average number of nodes of each annulus-sector in the *k*-th annulus, and the area of the *k*-th annulus is (2*k* − 1)*π*(*R*/*k*)^2^. Thus, it is easy to know that:

(8) $$N_{k}\approx \left(1/n\right)\left(2k-1\right)\pi {\left(R/k\right)}^{2}\rho$$

By combining Equations (7) and (8), we get:

(9) $$E_{CH}^{k}\approx l\left(\left(\left(\frac{2k-1}{n}\right){\left(\frac{R}{k}\right)}^{2}\pi \rho -1\right)e_{r}+\left(\frac{2k-1}{n}\right){\left(\frac{R}{k}\right)}^{2}\pi \rho e_{t}\right)$$

As for the CH in the *i*-th annulus (*i* ∈ [1, *k* − 1]), it not only needs to receive data uploaded by the non-CHs in this cluster, but also receives data forwarded by the corresponding CH in the (*i +* 1)-th annulus. Then, it sends all these data to one of the CHs in the (*i* − 1)-th annulus (when *i* ∈ [2, *k* − 1]) or to BS (when *i* = 1). The total number of nodes in the *i*-th annulus (*i* ∈ [*i +* 1, *k*]) is $\sum_{m=i+1}^{k}N_{m}$. Therefore, during a round of data collection, the energy consumption of this CH is:

(10) $$E_{CH}^{i}=l\left(\left(N_{i}-1+\sum_{m=i+1}^{k}N_{m}\right)e_{r}+\left(N_{i}+\sum_{m=i+1}^{k}N_{m}\right)e_{t}\right)$$

Similarly, *N_i_* is defined as the average number of nodes of each annulus-sector in the *i*-th annulus, so:

(11) $$N_{i}\approx \left(1/n\right)\left(2i-1\right)\pi {\left(R/k\right)}^{2}\rho$$

Also, according to Equations (10) and (11), it is known that:

(12) $$E_{CH}^{i}\approx l\left(\left(\left(\frac{k^{2}-{\left(i-1\right)}^{2}}{n}\right){\left(\frac{R}{k}\right)}^{2}\pi \rho -1\right)e_{r}+\left(\frac{k^{2}-{\left(i-1\right)}^{2}}{n}\right){\left(\frac{R}{k}\right)}^{2}\pi \rho e_{t}\right)$$

### 4.3. The Length of Radius of the RCCH

As mentioned above, the load on the CHs that is close to the center of the network is relatively higher although the area of the annulus-sector in which it is located is relatively smaller. It needs to forward a large amount of data uploaded by the CHs in the outer annuli. Therefore, for annulus-sectors in different annuli, the sizes of their RCCH should be different to balance the energy consumption of the whole network. In order to ensure the lifetime of each RCCH is approximately equal to each other, the following constraint needs to be met:

(13) $$\pi r_{1}^{2}\rho e_{0}/E_{CH}^{1}\approx \pi r_{2}^{2}\rho e_{0}/E_{CH}^{2}\approx \ldots \approx \pi r_{i}^{2}\rho e_{0}/E_{CH}^{i}\approx \pi r_{k}^{2}\rho e_{0}/E_{CH}^{k}$$

In (13), *πr_i_*^2^*ρe*_0_ is the total initial energy of the nodes in one RCCH of the *i*-th annulus. After simplification, that is:

(14) $$r_{i}/r_{i+1}\approx \sqrt{E_{CH}^{i}/E_{CH}^{i+1}}$$

When *i* ∈ [1, *k* − 2], according to Equations (10) and (14), it can be obtained that:

(15) $$\frac{r_{i}}{r_{i+1}}\approx \sqrt{1+\left(\left(N_{i}e_{r}+N_{i}e_{t}\right)/\left(\left(\sum_{m=i+1}^{k}N_{m}-1\right)e_{r}+\sum_{m=i+1}^{k}N_{m}e_{t}\right)\right)}$$

Since *N_i_* is always less than *N_i_*_+ 1_, the ratio between *r_i_* and *r_i_*_+ 1_ is larger than 1 but smaller than 2. That is, the closer the RCCH is to the network center, the larger its area is. When *i* = *k* − 1, after substituting Equation (10) into Equation (14), it can be obtained that:

(16) $$\frac{r_{k}}{r_{k-1}}\approx \sqrt{\frac{\left(N_{k}-1\right)e_{r}+N_{k}e_{t}}{\left(N_{k-1}+N_{k}-1\right)e_{r}+\left(N_{k-1}+N_{k}\right)e_{t}}}$$

According to Equation (11), it is known that *N_k−_*_1_ = (2*k* − 3)/(2*k* − 1) *N_k_*. By combining this equation and Equation (16), we can get:

(17) $$\frac{r_{k}}{r_{k-1}}\approx \sqrt{\left(\left(N_{k}-1\right)e_{r}+N_{k}e_{t}\right)/\left(\left(\left(\frac{4k-4}{2k-1}\right)N_{k}-1\right)e_{r}+\left(\frac{4k-4}{2k-1}\right)N_{k}e_{t}\right)}$$

It is not difficult to know that the ratio between *r_k_* and *r_k_*_−1_ is smaller than 1, so the area of the RCCH in the outermost annulus-sectors is the smallest as the CHs in these annulus-sectors need not forward data from other annulus-sectors. In this case, the burdens on these CHs are relatively light so there is no need for too many nodes to participate in CH selection.

According to Equation (13), the length of radius of the RCCH in the (*k* − 1)-th annulus is important for balance of energy consumption between CHs, because it affects not only the value of *r_k_*, but also the value of *r_i_* (*i* ∈ [1, *k* − 2]). For the annulus-sector in the *k*-th annulus, there must be at least one node in its RCCH. When *πr_k_*^2^*ρ* > 1, the above constraint can be satisfied. That is to say, the following formula must be met:

(18) $$r_{k}\geq 1/\sqrt{\pi \rho }$$

**Figure 5:**
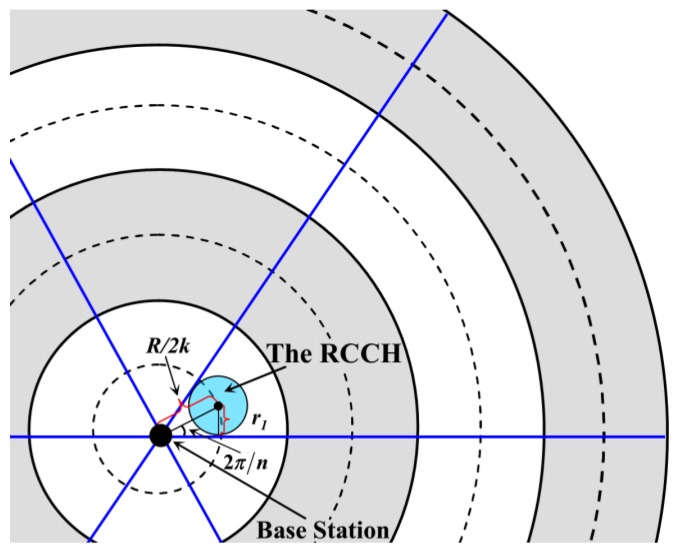
The maximal value of *r*_1_.

On the other hand, the range of the RCCH should not go beyond the boundary of the annulus-sector at which it is located. There is no doubt that the area of the annulus-sector in the first annulus is the smallest, and according to Equations (15) and (17), it is also known that the area of the RCCH in this annulus is the largest. Thus, we only care about the upper limit of *r*_1_. As shown in Figure 5, if and only if the boundaries of the RCCH and the annulus-sector are tangent, the value of *r*_1_ is the maximum, so it needs to meet the following expression:

(19) $$r_{1}\leq \left(R/2k\right)\sin\left(\pi /n\right)$$

According to Equations (15), (16), (18) and (19), the following equation can be obtained:

(20) $$r_{k-1}\geq \sqrt{\frac{\left(N_{k-1}+N_{k}-1\right)e_{r}+\left(N_{k-1}+N_{k}\right)e_{t}}{\pi \rho \left(\left(N_{k}-1\right)e_{r}+N_{k}e_{t}\right)}}$$

Similarly, according to Equations (15) and (19), the following expression can be obtained:

(21) $$r_{k-1}\leq \sqrt{\frac{\left(N_{k-1}+N_{k}-1\right)e_{r}+\left(N_{k-1}+N_{k}\right)e_{t}}{\left(\left(N_{1}-1+\sum_{m=2}^{k}N_{m}\right)e_{r}+\left(N_{1}+\sum_{m=2}^{k}N_{m}\right)e_{t}\right)}}\times \left(\frac{R}{2k}\right)\sin\left(\frac{\pi }{n}\right)$$

### 4.4. The Length of the Communication Radius of Sensor Node

Obviously, in AEBDC, it is necessary to ensure that each non-CH can upload data to the CH within one hop. As mentioned above, the area of the annulus-sector in the *k*-th annulus is the largest. Therefore, one only needs to ensure that the distance between any node and the CH in this annulus-sector is no longer than the length of the communication radius (denoted as *r_t_*).

**Figure 6:**
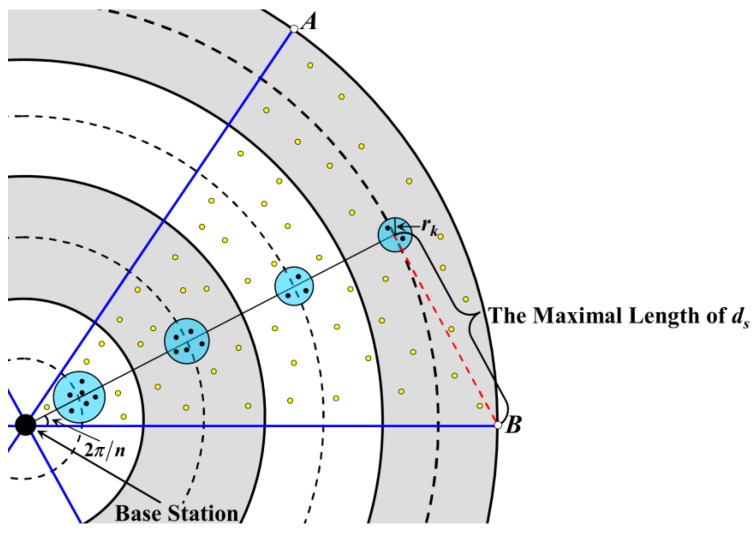
Analysis on the length of the communication radius.

Like in Section 4.2, the Euclidean distance from any node in the *k*-th annulus to the center of its RCCH is defined as *d_s_*. As shown in Figure 6, the value of *d_s_* is maximal if and only if this node is located at point A or B. In this case, the maximum value is:

(22) $$d_{s}=\sqrt{R^{2}+{\left(2k-1\right)}^{2}{\left(R/2k\right)}^{2}-\left(2k-1\right)\left(R^{2}/k\right)\cos\left(\pi /n\right)}$$

Moreover, no matter where the cluster header is located at, the node in the same annulus-sector should be able to upload data to it within one hop. Thus, the following requirement should also be met:

(23) $$r_{t}\geq d_{s}+r_{k}$$

On the other hand, the CHs in two adjacent annulus-sectors of two different annuli should also be able to communicate with each other within one hop to ensure that data can eventually be uploaded to BS. According to Section 4.3, the closer the annulus-sector is to the BS, the larger the area of its RCCH is, so it is not difficult to know that the furthest distance between two communicable CHs is equal to the distance from *c*_1_ (the CH in the first annulus) to *c*_2_ (the CH in the second annulus), as shown in Figure 6. That is to say:

(24) $$r_{t}\geq \left(R/k\right)+r_{1}+r_{2}$$

In summary, the value of *r_t_* should meet the following equation:

(25) $$r_{t}\geq \mathrm{Max}\left(d_{s}+r_{k},\;\left(R/k\right)+r_{1}+r_{2}\right)$$

### 4.5. Data Forwarding Strategy with the Help of the Candidate Cluster Heads

It is obvious that in the annulus-sector of the *k*-th annulus, the number of nodes is the most, so o, in order to ensure that the buffer of the CH in the *k*-th annulus (i.e., *c_k_*) does not overflow, the following requirement should be met during a round of data gathering. The buffer size is set to *C*′:

(26) $$l\times N_{k}\leq C\prime$$

In order to make full use of the storage space, the left and right side of Equation (26) are made equal in this paper. That is, the total amount of data collected by *c_k_* is exactly equal to its buffer size at the end of a round of data collection.

**Figure 7:**
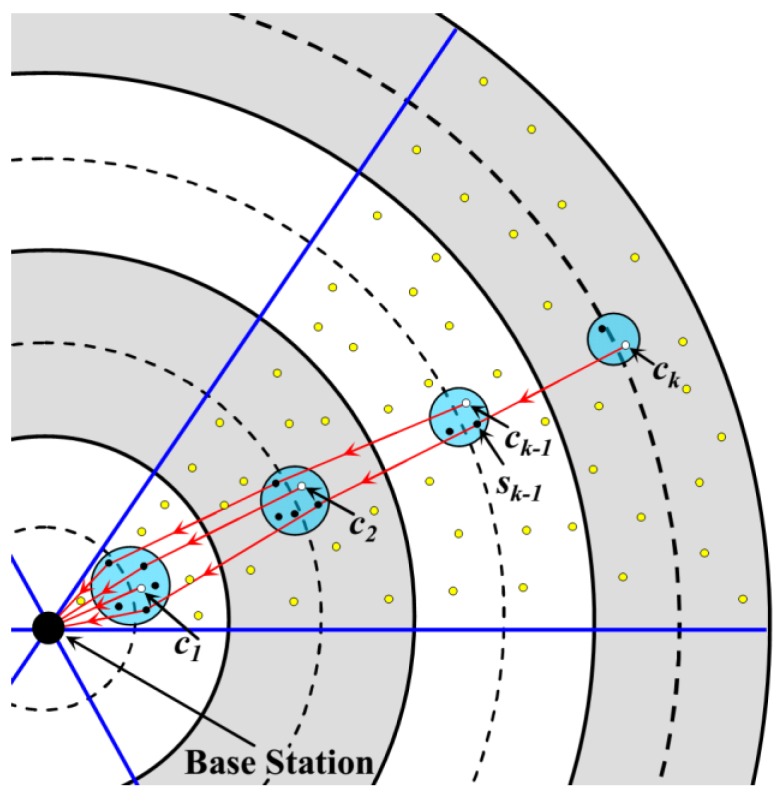
Data forwarding strategy with the help of the candidate cluster heads.

However, if *c_k_* sends the *C*′ bits of data directly to *c_k_*_−1_ (the CH in the (*k* − 1)-th annulus) at this time, the buffer of *c_k_*_−1_ will overflow as *l × N_k_*_−1_ bit of data has been stored. However, the buffer of each CCH in the RCCH where *c_k_*_−1_ is located at is empty right now. Thus, the CCH with the highest residual energy is selected as the next-hop receiver of *c_k_*. That is to say, in each RCCH of the (*k* − 1)-th annulus, there are two nodes responsible for data uploading. One is the CH (i.e., *c_k_*_−1_ in Figure 7, and another one is the CCH (i.e., node *s_k_*_−1_ in Figure 7. Similarly, in each RCCH, one CCH should be selected to receive and forward the data uploaded by the CH in the adjacent outer annulus. Therefore, for the RCCH in the *i*-th annulus, there are *k* − *i* + 1 nodes (one CH and *k* − *i* CCHs) need to send data to the corresponding nodes in the (*i* − 1)-th annulus, as shown in Figure 7. The specific steps of the data forwarding strategy are described as follows:

(1) For the RCCH in the *i*-th annulus (*i < k*), it is assumed that the nodes which are responsible for data forwarding have already been selected. The cluster head is defined as *c_i_*, and the other *k* − *i* forwarding nodes are denoted as *c_i_*_,1_, *c_i_*_,2_, …, *c_i_*_,*k*−*i*_, respectively, according to the load on them from high to low.
(2) Then, *c_i_* sends the “request for data forwarding” message to all the CCHs in the adjacent annulus-sectors of the *i* − 1-th annulus.
(3) After receiving this message, these CCHs return the reply messages with their residual energy information to *c_i_*, respectively.
(4) After receiving all the reply messages, those *k* − *i +* 1 CCHs with the most residual energy are selected as the forwarding nodes. According to the residual energy ranking of them from high to low, they are denoted as *c_i−_*_1,1_, *c_i−_*_1,*2*_, *…*, *c_i_*_−1,*k*−*i*+1_. Then, *c_i_*_-1,1_, *c_i_*_-1,2_, …, *c_i−_*_1,*k−*1_ become the next-hop receivers of *c_i_*_,2_, …, *c_i_*_,*k*−*i*_ respectively, while *c_i−_*_1,*k−i+*1_ is the next-hop receiver of *c_i_*.

Thus, with the help of the CCHs, data packet loss due to buffer overflow could be avoided. In addition, the energy consumption of the CH and CCH could also be balanced to a certain extent.

It is worth mentioning that, to implement this data forwarding strategy, the number of nodes in the RCCH of the (*i* − 1)-th annulus should be one more than those in the RCCH of the *i*-th annulus. According to Equation (15), it is known that this constraint can be met.

In addition, it is not difficult to know from Equation (17) that if there are few nodes in the RCCH of the *k*-th annulus, in order to ensure the success of data forwarding, the value of *k* should be restricted. Considering the worst case, when there is only one node in the RCCH of the *k*-th annulus, the number of nodes in the RCCH of the (*k* − 1)-th annulus should be no less than two. That is to say, the following formula should be met:

(27) $$r_{k-1}{}^{2}/r_{k}{}^{2}\geq 2$$

According to Equations (17) and (27), we have:

(28) $$\frac{\left(\left(\frac{4k-4}{2k-1}\right)N_{k}-1\right)e_{r}+\left(\frac{4k-4}{2k-1}\right)N_{k}e_{t}}{\left(N_{k}-1\right)e_{r}+N_{k}e_{t}}\geq 2$$

When *k* > 4, it can be approximately regarded that the constraint about in Equation (28) could be met. Therefore, under the condition that the density of nodes in the network is low, the number of annuli should be more than 4.

## 5. The Recharging Scheme for the Nodes in the RCCH

Although the efficiency of data transmission can be enhanced with the help of the nodes in the RCCH, the average energy consumption of CHs and CCHs are still higher than that of the CNs in the same annulus-sector. Due to this unbalanced energy consumption, an “energy hole” may appear.

In recent years, using one or more Wireless Charging Vehicles (WCVs) to recharge sensor nodes periodically or responsively has become a research hotspot. However, the application bottlenecks (e.g., slow wireless recharging speed, high recharging cost, etc.) have made it unrealistic to recharge every node in the network. 

For this purpose, it is assumed that in each annulus, there is a WCV that is responsible for recharging nodes in the RCCH of the same annulus. By minimizing the recharging cost, the energy consumption of the entire network may be balanced and the energy hole problem may be alleviated. The definitions of wireless recharging parameters are given in Table 3.

### 5.1. Wireless Recharging Model

**Figure 8:**
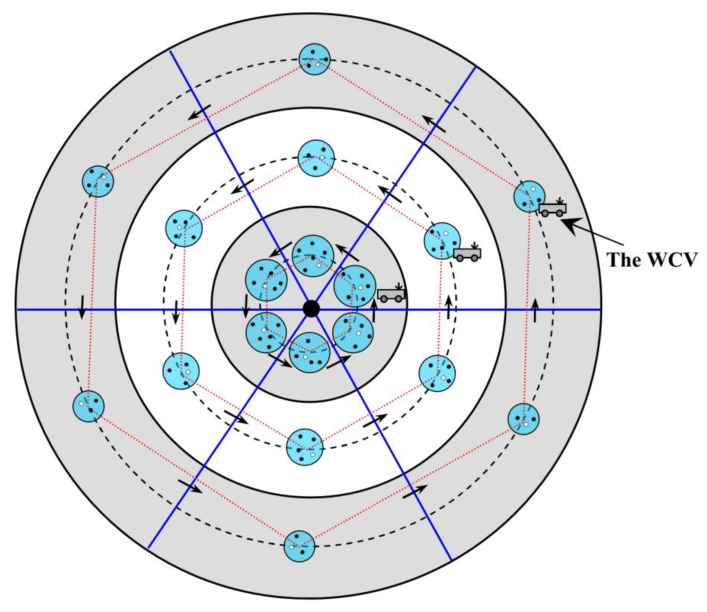
Multi-WCV-based wireless recharging scheme.

As shown in Figure 8, there is a WCV on the center line of each annulus, and the initial position of each WCV is the center of the first RCCH of each annulus. During the recharging process, each WCV only stays at the center of each RCCH and recharges the CH and CCHs wirelessly. After finishing the recharging task of one RCCH, it moves along a straight line to the next RCCH for recharging, and finally it returns to the initial position.

Taking the WCV in the *i*-th annulus as an example, the wireless recharging strategy is described as follows. *T_i_^r^* is defined as a round of recharging time, that is the time duration from the moment the WCV leaves from the initial position to the moment it returns back again. In addition, *t_ij_^c^* is defined as the time duration for the WCV to recharge nodes in the *j*-th RCCH of the *i*-th annulus. Moreover, the time spending on moving from the *j*-th RCCH to the (*j +* 1)-th RCCH is denoted as *t_ij_^m^*. Thus:

(29) $$T_{i}^{r}=\sum_{j=1}^{n}\left(t_{ij}^{c}+t_{ij}^{m}\right)$$

and, the total moving path length of WCV during *T_i_^r^* is:

(30) $$l_{i}=n\left(2i-1\right)\left(R/k\right)\sin\alpha$$

The recharging scheduling strategy in this paper should mainly solve the following two problems:

How to set the most appropriate energy threshold of the recharging request?
How to balance the energy consumption of nodes as much as possible while maximizing the recharging efficiency?

The detailed analysis is as follows.

### 5.2. Energy Threshold of the Recharging Request

As mentioned above, the CH election is carried out after each round of data gathering. Therefore, for the convenience of analysis, the total residual energy of all nodes in the same RCCH is taken as a whole. Moreover, nodes are uniformly and randomly distributed in the circular network, and the energy consumption on nodes in the same RCCH could be balanced due to the collaboration-based data forwarding strategy. Thus, it is regarded that the energy consumption rates of each RCCH are approximately equal to each other. When receiving the recharging request from the first RCCH, the WCV starts the first round of recharging. If the residual energy of WCV is exactly zero when it returns back to the initial position at the end of the first round of recharging, the recharging benefits reaches to the maximal value. In this case, the following equation is met:

(31) $$\sum_{j=1}^{n}R_{i,j}^{\left(1\right)}+l_{i}P_{m}=B$$

In (31), $\sum_{j=1}^{n}R_{i,j}^{\left(1\right)}$ is the total amount of energy that WCV recharges for nodes in all the RCCH of the *i*-th annulus during the first round of recharging, and *l_i_P_m_* is the total energy consumption of WCV when moving in the *i*-th annulus. At the same time, for the nodes in the last RCCH (the *n*-th RCCH), it needs to ensure that they still alive when the WCV arrives. Thus, for this region, the minimum value of the total energy that ensures nodes alive is defined as *E_i_^min^.*

On the other hand, it is easy to know that the CNs in the *k*-th annulus have the highest energy consumption rate among all the non-rechargeable nodes as the average data transmission distance of them is the longest, so if the average residual energy of nodes in a RCCH one moment before recharging is equal to that of the CNs in the *k*-th annulus at the same moment, it is regarded that the balance of energy consumption is achieved. Therefore, when the WCV arrives at the *j*-th annulus-sector of the *i*-th annulus the second time, the following equation should be met:

(32) $$\frac{R_{i,j}^{\left(1\right)}+E_{i,j}^{r\left(1\right)}-\left(T_{i}^{r}/t_{r}\right)E_{CH}^{i}}{\pi r_{i}^{2}\rho }=\frac{E_{i,j}^{r(1)}\left(k\right)-\left(T_{i}^{r}/t_{r}\right)E_{CN}^{k}}{N_{k}-\pi r_{k}^{2}\rho }$$

In (32), (*T_i_^r^*/*t_r_*)/*E_CH_^i^* is the total energy consumption of nodes in the *j*-th RCCH of the *i*-th annulus during the waiting time of recharging. Similarly, (*T_i_^r^*/*t_r_*)/*E_CN_^i^* is the total energy consumption of CNs in each annulus-sector of the *k*-th annulus during the waiting time of recharging. In addition, *πr_i_*^2^*ρ* is the average number of nodes in each RCCH of the *i*-th annulus. Thus, *N_k_* − *πr_i_*^2^*ρ* is the average number of CNs in each annulus-sector of the *k*-th annulus. Moreover, the value of $E_{i,j}^{r(1)}\left(k\right)$ can be further expressed as:

(33) $$E_{i,j}^{r(1)}\left(k\right)=E_{CN}^{k}\times \left(T_{i}^{r}-\sum_{j=1}^{j}\left(t_{ij}^{c}+t_{ij}^{m}\right)\right)/t_{r}+E_{i,n}^{r(1)}\left(k\right)$$

In (33), $E_{i,n}^{r(1)}\left(k\right)$ is defined as total residual energy of all the CNs in one annulus-sector of the *k*-th annulus at the moment when the WCV arrives at the *n*-th RCCH of the *i*-th annulus the first time. Meanwhile, the total residual energy of nodes in the *n*-th RCCH is approximately equal to *E_i_^min^* at this moment. When the total residual energy of nodes in the *n*-th RCCH is equal to *E_i_^min^*, the network has already run for $\left(\pi r_{i}^{2}\rho e_{0}-E_{i}^{\min}\right)/E_{CH}^{i}$ rounds. Therefore, it is not difficult to know that:

(34) $$E_{i,n}^{r(1)}\left(k\right)=\left(N_{k}-\pi r_{k}^{2}\rho \right)e_{0}-\left(\left(\pi r_{i}^{2}\rho e_{0}-E_{i}^{\min}\right)/E_{CH}^{i}\right)\times E_{CN}^{k}$$

By combining Equations (31)–(34), the value of $E_{i,j}^{r\left(1\right)}$ can be calculated. Without loss of generality, $E_{i,1}^{r\left(1\right)}$ is regarded as the energy threshold of the recharging request. In each annulus, if the total residual energy of nodes in one RCCH is lower than the threshold, a recharging request is send to the WCV, and then the first round of recharging begins.

### 5.3. The Amount of Energy Provided by the WCV in Each Round of Recharging

It is known from the above analysis that, for each node being recharged in the *i*-th annulus, the time interval between the first and the second recharging is *T_i_^r^.* Furthermore, the energy consumption rates of the common nodes are relatively stable, and the WCV moves at a constant speed. Thus, for different RCCHs of the same annulus, the energy supplemented from the WCV after the first round of recharging are the same in each round of recharging. To further optimize the recharging strategy of WCV, we analyze the waiting time of the rechargeable nodes.

**Figure 9:**
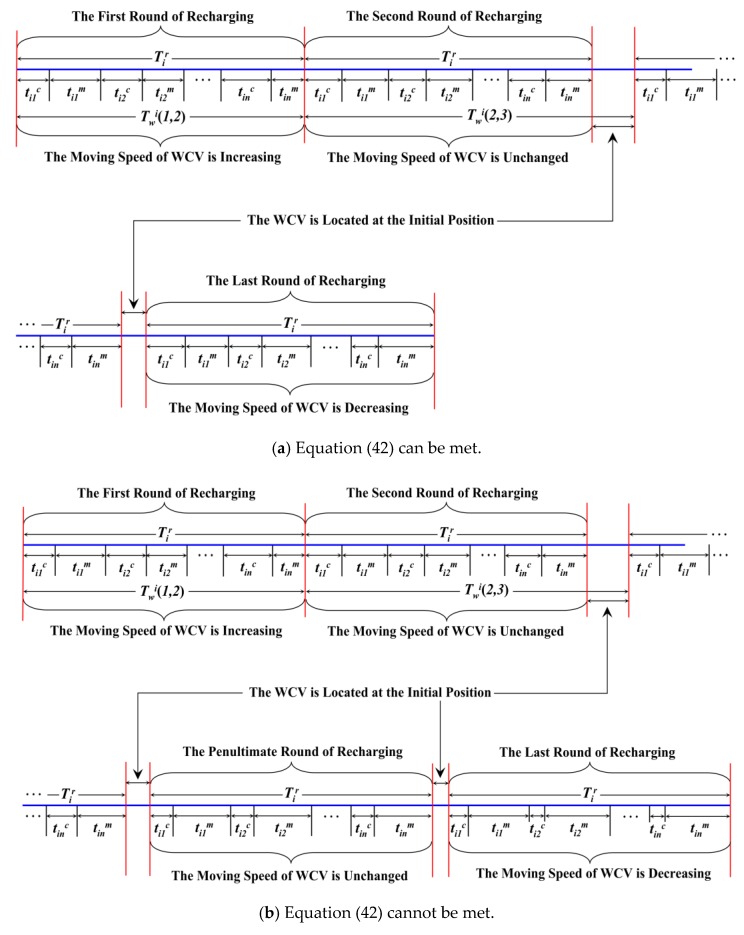
The AEBDC recharging sequence diagram.

*T_w_^i^*(*a*, *a +* 1) is defined as the waiting time of the rechargeable nodes in the *i*-th annulus from the end of the *a*-th round of recharging to the beginning of the (*a +* 1)-th round of recharging. Thus, *T_w_^i^*(1, 2) = *T_i_^r^*, as shown in Figure 9a. However, it is not reasonable if this waiting time is still *T_i_^r^* after the second round of recharging. In this case, the residual energy of the rechargeable nodes is still high, so there is no need to recharge it again for the time being. On the other hand, the frequent movement of WCV will consume more energy and also result in low recharging efficiency.

For this reason, we discuss the situation after the second round of recharging. To ensure that the average residual energy of nodes in a RCCH is equal to that of the CNs in the *k*-th annulus at the moment when the WCV arrives at this RCCH for the *a*-th time (*a ≥ 2*), Equation (35) should be met:

(35) $$E_{i,j}^{r\left(a\right)}/\pi r_{i}^{2}\rho =E_{i,j}^{r(a)}\left(k\right)/\left(N_{k}-\pi r_{k}^{2}\rho \right)$$

Similarly, when the WCV arrives at the same RCCH for the (*a +* 1)-th time, the above constraint still needs to be met. According to Equation (32), it is known that:

(36) $$\frac{R_{i,j}^{\left(a\right)}+E_{i,j}^{r\left(a\right)}-\left(T_{w}^{i}\left(a,a+1\right)/t_{r}\right)E_{CH}^{i}}{\pi r_{i}^{2}\rho }=\frac{E_{i,j}^{r(a)}\left(k\right)-\left(T_{w}^{i}\left(a,a+1\right)/t_{r}\right)E_{CN}^{k}}{N_{k}-\pi r_{k}^{2}\rho }$$

By substituting Equation (35) into (36), the amount of energy (denoted as *R_i_*_,*j*_^(*a*)^) that the WCV recharges for the nodes in this RCCH for the *a*-th time could be expressed as:

(37) $$R_{i,j}^{\left(a\right)}=\frac{T_{w}^{i}\left(a,a+1\right)\left(\left(N_{k}-\pi r_{k}^{2}\rho \right)E_{CH}^{i}-\pi r_{i}^{2}\rho E_{CN}^{k}\right)}{t_{r}\left(N_{k}-\pi r_{k}^{2}\rho \right)}$$

From Equation (37), we can find that the value of *R_i_*_,*j*_^(*a*)^ is unrelated to the value of *j*. It means that the amount of energy for recharging the nodes in each RCCH of the *i*-th annulus is unchanged after the second round of recharging. On the other hand, after finishing the *a*-th round of recharging, the residual energy of WCV should be enough to ensure that it can return back to its initial position. That is:

(38) $$B\geq R_{i,j}^{\left(a\right)}\times n+p_{m}l_{i}$$

so, the value of *R_i_*_,*j*_^(*a*)^ needs to meet Equation (39):

(39) $$R_{i,j}^{\left(a\right)}\leq \left(B-p_{m}l_{i}\right)/n$$

In addition, to avoid energy waste, it is necessary to ensure that there is no “overcharge”. That is, the energy recharging for a node plus its residual energy should not be greater than its maximum battery capacity. It is not difficult to conclude that, during each round of recharging, when the WCV arrives at the first RCCH, the average residual energy of nodes in this RCCH is higher than that of the nodes in other RCCHs when the WCV arrives at them. Therefore, it only need to ensure that the nodes in the first RCCH do not overcharged. That is:

(40) $$R_{i,1}^{\left(a\right)}\leq \pi r_{i}^{2}\rho e_{0}-E_{i,1}^{r\left(a\right)}$$

According to Equations (37), (39) and (40), the value of *R_i_*_,*j*_^(*a*)^ can be calculated.

### 5.4. The Number of Recharging Rounds

In this paper, it is regarded that when the node’s residual energy is less than 5% of its initial energy, it is dead. Thus, at the beginning of the (*a +* 1)-th round of recharging, the WCV will judges whether or not one or more CNs in the *k*-th annulus will die at the end of this round of recharging. That is to say, we need to judge whether Equation (41) is met:

(41) $$E_{i,j}^{r\left(a\right)}\left(k\right)-E_{CN}^{k}\times \left(T_{w}^{i}\left(a,a+1\right)/t_{r}\right)\geq 5\%\times e_{0}\times \left(N_{k}-\pi r_{k}^{2}\rho \right)$$

*E^k^_CN_* × (*T_w_^i^*(*a*, *a +* 1)/*t_r_*) is the total energy consumption of CNs in the *k*-th annulus during *T_w_^i^*(*a*, *a +* 1).

By combining Equations (37), (39) and (40), we can get the upper limit value of *T_w_^i^*(*a*, *a +* 1), and it is then substituted into the left side of Equation (41).

Case 1. If Equation (41) is met, it can predict that when the WCV finishes the (*a +* 1)-th round of recharging, no common nodes die. Therefore, the upper limit value of *T_w_^i^(a*, *a +* 1) is regarded as the waiting time of the rechargeable nodes between the *a*-th round and the (*a* + 1)-th round of recharging.
Case 2. If Equation (41) cannot be met, the maximal value of *T_w_^i^(a*, *a +* 1) that satisfies Equation (41) is taken as the waiting time. In this case, the total energy recharging for nodes in one RCCH is less than *(B − p_m_l_i_)*/*n* in the (*a +* 1)-th round of recharging. Meanwhile, the left side of Equation (41) equals the right side of it. Therefore, when the WCV arrives at the last RCCH in the (*a +* 1)-th round of recharging, one or more common nodes will die and the whole network lifetime ends. Of course, the WCV will not recharge the last RCCH.

In Case 2, it just ensures that all the nodes in each RCCH of the *i*-th annulus are almost dead at the same time when the WCV arrives at the last RCCH in the (*a +* 1)-th round of recharging. Therefore, the efficiency of the (*a +* 1)-th round of recharging is obviously not high enough. In this case, we add the amount of energy recharging for nodes in the (*a +* 1)-th round to the *a*-th round, and cancel the (*a +* 1)-th round of recharging. Moreover, it is still necessary to judge whether the “overcharge” occurs in the RCCH of the first annulus-sector. In other words, we need to judge whether Equation (42) is met:

(42) $$R_{i,1}^{\left(a\right)}+\frac{\left(n-j\right)\left(t_{ij}^{c}+t_{ij}^{m}\right)\times \left(\left(N_{k}-\pi r_{k}^{2}\rho \right)E_{CH}^{i}-\pi r_{i}^{2}\rho E_{CN}^{k}\right)}{t_{r}\left(N_{k}-\pi r_{k}^{2}\rho \right)}\leq \frac{B-p_{m}l_{i}}{n}$$

According to Equation (37), it is not difficult to know that, $\frac{\left(n-j\right)\left(t_{ij}^{c}+t_{ij}^{m}\right)\times \left(\left(N_{k}-\pi r_{k}^{2}\rho \right)E_{CH}^{i}-\pi r_{i}^{2}\rho E_{CN}^{k}\right)}{t_{r}\left(N_{k}-\pi r_{k}^{2}\rho \right)}$ refers to the total amount of energy recharging for nodes of the first RCCH in the (*a +* 1)-th round of recharging. If Equation (42) is met, the WCV only needs to do *a* rounds of recharging, otherwise it must carry out the (*a +* 1)-th round of recharging.

### 5.5. The Moving Speed of WCV

From Section 5.2 to Section 5.4, it can be seen that the total amount of energy recharging for nodes is not always the same in different rounds. Moreover, the value of *T_i_^r^* is unchanged during the network lifetime, so the value of *v_WCV_* needs to be discussed in this Section. It is assumed that the movement of a WCV from one RCCH to another is a uniform linear motion. When *a* = 1, according to Equation (32), it is known that:

(43) $$R_{i,j}^{\left(a\right)}=\left(\frac{E_{i,j}^{r(1)}\left(k\right)-\left(T_{i}^{r}/t_{r}\right)E_{CN}^{k}}{N_{k}-\pi r_{k}^{2}\rho }\right)\pi r_{i}^{2}\rho +-\left(T_{i}^{r}/t_{r}\right)E_{CH}^{i}-E_{i,j}^{r\left(1\right)}$$

When a > 1,

- If Equation (41) is met, the value of $R_{i,j}^{\left(a\right)}$ should be calculated by Equation (37).
- If Equation (41) cannot be met but Equation (42) is met, the right expression of Equation (37) should be substituted into Equation (42). In this case, the *a*-th round of recharging is the last round, and the value of $R_{i,j}^{\left(a\right)}$ is:
  (44) $$R_{i,j}^{\left(a\right)}=\left(T_{w}^{i}\left(a,a+1\right)+\left(n-j\right)\left(t_{ij}^{c}+t_{ij}^{m}\right)\right)\times \frac{\left(\left(N_{k}-\pi r_{k}^{2}\rho \right)E_{CH}^{i}-\pi r_{i}^{2}\rho E_{CN}^{k}\right)}{t_{r}\left(N_{k}-\pi r_{k}^{2}\rho \right)}$$
- If both Equations (41) and (42) are not met, the amount of energy recharging for each RCCH in the *a*-th round is calculated by Equation (37), and the amount of energy recharging for each of them in the (*a +* 1)-th round is:
  (45) $$R_{i,j}^{\left(a+1\right)}=\frac{\left(n-j\right)\left(t_{ij}^{c}+t_{ij}^{m}\right)\times \left(\left(N_{k}-\pi r_{k}^{2}\rho \right)E_{CH}^{i}-\pi r_{i}^{2}\rho E_{CN}^{k}\right)}{t_{r}\left(N_{k}-\pi r_{k}^{2}\rho \right)}$$

As shown in Figure 9b, the (*a +* 1)-th round of recharging is the last round, while in Figure 9a, the last round is the *a*-th round of recharging, which is one round less than the case in Figure 9b.

According to Equation (43), it is easy to see that during the first round of recharging, the amount of energy recharging for each RCCH increases with the increase of *j*. Thus, the value of *t_ij_^c^* is also increasing. However, the value of *T_i_^r^* is always unchanged, so the moving speed of WCV on different segments should be increased one by one. Moreover, the moving path length of WCV is equal between any two adjacent RCCHs in the same annulus. Thus, in Figure 9a, the length of *t_ij_^m^* decreases with the increase of *j* in the first round of recharging.

In addition, it can be seen from Equation (37) that from the second round of to the penultimate round of recharging, the amount of energy recharging for each RCCH is equal to each other in the same round. Therefore, the value of *v_WCV_* on different segments should be the same in this case, and the length of *t_ij_^m^* shown in Figure 9a is unchanged after the first round of recharging.

From Equations (44) and (45), it also knows that in the last round of recharging, the amount of energy recharging for each RCCH is constantly decreasing. Furthermore, the value of *t_ij_^c^* is also decreasing with the increase of *j*. Thus, the moving speed of WCV on different segments should be gradually decreased. 

In summary, the relationship among the moving speed of WCV, the amount of energy for recharging and the time for one round of recharging can be expressed as follows:

(46) $$R_{i,j}^{\left(a\right)}/P_{c}+\left(l_{i}/n\right)/v_{WCV}=T_{i}^{r}/n$$

That is:

(47) $$v_{WCV}=P_{c}l_{i}/n\left(P_{c}T_{i}^{r}-R_{i,j}^{\left(a\right)}\right)$$

From Equation (47), it is easy to know that the value of *v_WCV_* increases with the increase of $R_{i,j}^{\left(a\right)}$ which is relevant to *t_ij_^c^*. Therefore, the variation of *v_WCV_* is relevant to the recharging time *t_ij_^c^*, which is consistent with the above analysis. Figure 9 is the sequence diagram about each round of recharging.

## 6. Simulation Results and Analysis

To verify the performance of AEBDC in terms of energy consumption balance, network lifetime and recharging efficiency, relevant experiments were carried out with the help of Java (JDK1.8) and Matlab R2014a. These simulation results were compared with EBCAG [16] and EBCH [17], which are two cluster-based data collection strategies in WSNs. Values of the experimental parameters are shown in Table 4.

### 6.1. Energy Consumption of Nodes under Different Kinds of Network Partition

**Figure 10:**
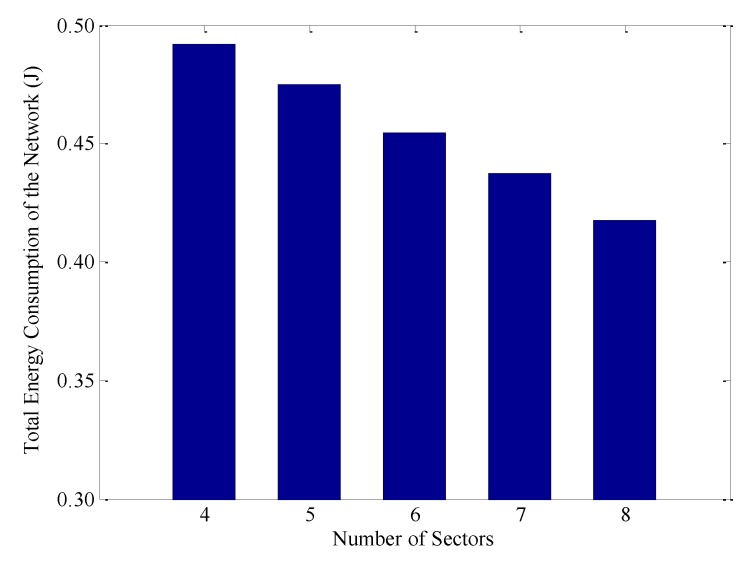
Total energy consumption under different numbers of sectors in a round of data collection.

Figure 10 shows the total energy consumption of nodes after a round of data collection under different numbers of sectors. Without loss of generality, the number of annuli is set to 3, that is, *k* = 3. Moreover, according to Equation (21), it is not difficult to know that, to ensure that there is at least one node in each RCCH of the outermost annulus, the network can be divided into eight sectors at most, namely *n* ≤ 8. Obviously, with the increase of the value of *n*, the total energy consumption of the whole network is decreasing. For example, when *n* = 8, the energy consumption is 17.8% lower than that at *n* = 4. The main reason is that the area of each annulus decreases with the increase of *n*, which releases the burden of nodes in the RCCH to some extent and decreases their energy consumption. In addition, the distance between the CN and the CH is also shortened when *n* is large. Thus, the energy consumption on single-hop transmission is also effectively reduced.

Then, we keep the network scale unchanged and analyze the total energy consumption under different kinds of network partition methods. The radius of the circular network is set to 240 m, and the total number of nodes in the network is 500. According to Equations (20) and (21), the network can be divided into five annuli at most in order to ensure that there is at least one node in each RCCH. Thus, we adopt three kinds of division, namely, *k* = 3 and *n* = 8; *k* = 4 and *n* = 5 and *k* = 5 and *n* = 4. Table 5 shows the average number of nodes in each RCCH under these three partition methods as well as the total energy consumption of them in one round of data collection. We can find that the energy consumption is the lowest when *k* = 3 and *n* = 8, which is respectively 75% and 65% of that in other two modes. It further illustrates the conclusion that “the smaller the annulus-sector is, the lower the total energy consumption will be”. It is worth noting that when the number of annulus-sectors is 20, the energy consumption of the network with *k* = 4 and *n* = 5 is about 0.0916 J lower than that with *k* = 5 and *n* = 4. This is because the area of the innermost RCCH of the former is much larger than that of the latter. Thus, more nodes can participate in data transmission. In addition, the fewer the number of annuluses is, the fewer the hops is, that results in lower energy consumption on communication.

**Figure 11:**
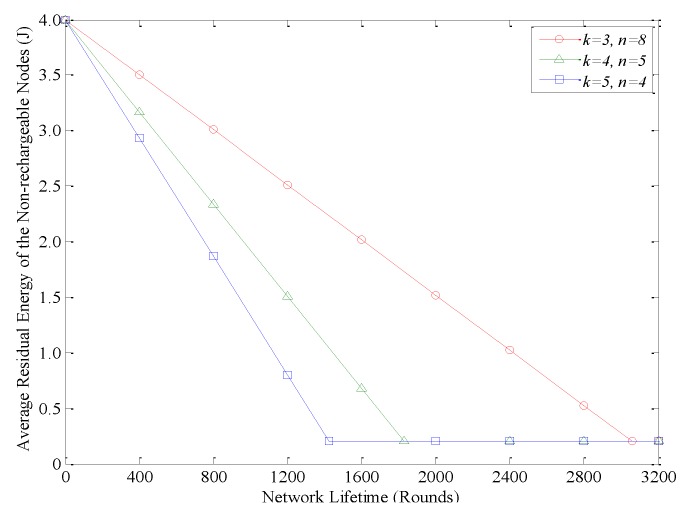
Average residual energy of the non-rechargeable nodes in the outermost annuli.

As mentioned above, in AEBDC, the WCV periodically recharges nodes located in the RCCH to ensure their sustainable operation. Compared with other non-rechargeable nodes, those nodes in the outermost annuli have the highest energy consumption rate due to their long single-hop transmission distance. For this reason, we focus on the average residual energy of those nodes, and the result is shown in Figure 11. When *k* = 3 and *n* = 8, the performance of this experiment is the best, and the dead nodes only appear after about 3000 rounds. However, in the case of *k* = 4, *n* = 5 or *k* = 5, *n* = 4, the dead nodes appear after 1800 or 1400 rounds, respectively.

Table 6 shows the average energy consumption of nodes in each RCCH during a round of data collection. No matter how many annuli are in the network, the energy consumption of nodes in different RCCHs within the same annulus is approximately equal to each other. This is because the cluster head is periodically selected in AEBDC and it also makes full use of the non-cluster heads with high residual energy and low load to forward data collaboratively. This effectively balances the energy consumption of nodes near the center of each annulus-sector. It is worth noting that in Table 6, the energy consumption balance at *k* = 3 is slightly worse than that at *k* = 4 or *k* = 5. The reason is that when the network is divided into three annuli, according to Equation (17), the area of RCCHs in the second and the third annulus are almost the same. Due to the uniform distribution of nodes, the number of nodes in these RCCHs are almost the same. However, the load on the nodes in the second annulus is heavier than those in the third annulus, thus, there is a certain difference in their energy consumption.

### 6.2. The Recharging Efficiency under Different Kinds of Network Partition

**Figure 12:**
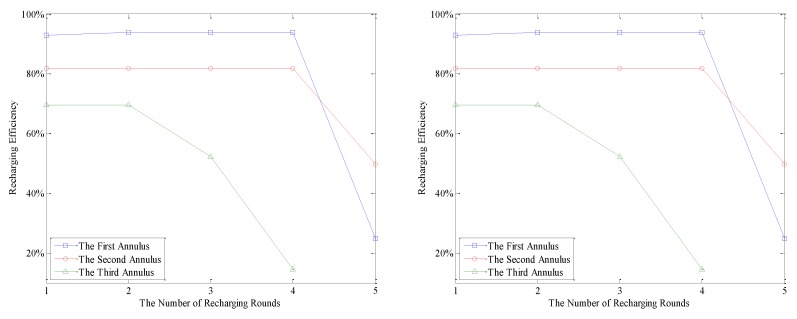

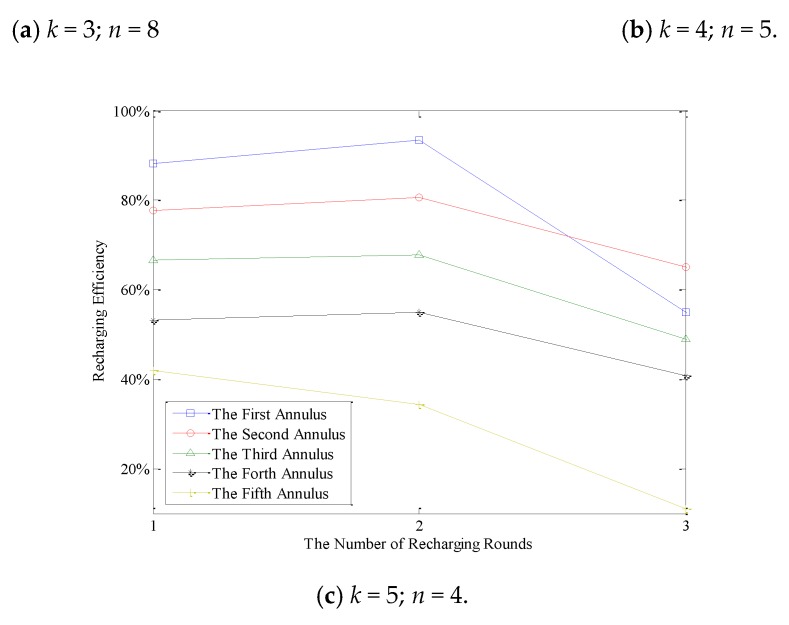
The recharging efficiency of WCV in each annulus (*k* = 3 and *n* = 8).

In AEBDC, the “recharging efficiency” is defined as the ratio between the total amount of energy recharged for nodes in a round of recharging and the battery capacity of WCV. From Figure 12, it is not difficult to see that no matter what the value of *n* and *k* is, the recharging efficiency of WCV in each annulus is relatively high except for the last one or two rounds. For example, in the case of *k* = 3 and *n* = 8, the recharging efficiency of WCV in the innermost annulus is still higher than 90% during the first three recharging cycles. In this case, the energy consumption rates of nodes in each RCCH are basically stable, so the most appropriate recharging request thresholds of them can be accurately calculated out by Equations (31)–(34). Furthermore, according to Equations (37), (39) and (40), the total amount of energy recharged for nodes in each RCCH during a round of recharging can also be calculated. Therefore, the energy that the WCV carries before each recharging is made full use of.

It can also be found from Figure 12 that the closer the WCV is to the center of the network, the higher recharging efficiency it has, regardless of the network partition modes. In this case, the moving path of the WCV is shorter than those of the other annuluses, so the energy consumption on moving is lower. Furthermore, the RCCHs that close to the center of the network consume more energy, so the amount of energy recharged for nodes is also slightly higher. It can also be found in this figure that, the recharging efficiency of the last two rounds decreases greatly. As mentioned before, in AEBDC, the moment when the first dead node appears in the outermost annulus is regarded as the end of the network lifetime. Therefore, in order to avoid energy waste, all nodes are do not need to be fully recharged in the last one or two rounds. The detailed recharging strategy has been described in Section 5.4.

It should be pointed out that we try to make the values of the various parameters in this paper as close as possible to that in the real scene. As is known to all, the development of current wireless recharging technology is still at an initial stage and the recharging rate is relatively slow. Therefore, there are not many recharging cycles during the simulation process.

Table 7 shows the total recharging efficiency and WCV’s energy distribution under the three kinds of network partition modes. It can be seen that when *k* = 3 and *n* = 8, the total recharging efficiency of WCV is the highest, while in other two cases, there is little difference between the total recharging efficiency.

### 6.3. Comparison of Network Lifetime

In this subsection, we compare our method with EBCAG and EBCH. In AEBDC, the network is divided into several virtual concentric annuluses with the same width, which is the same as EBCAG and EBCH. The nodes are organized into uneven clusters in these three methods for balance of energy consumption.

It is worth mentioning that in AEBDC, we use wireless charging technology, which is not adopted in EBCAG and EBCH. At present, in most wireless sensor network recharging strategies, it is regarded that all the nodes are rechargeable. So, the purpose of this kind of recharging schemes is to make the network run stably for a long time, and even there will never be dead nodes. However, the cost of recharging has not been fully taken into account, and some researchers even regard that the nodes can continuously obtain energy. This is obviously not practical. In AEBDC, we mainly focus on energy balance technology in Wireless Sensor Networks. The recharging strategy is only one of the means to improve the energy balance of the whole network. Thus, in the proposed algorithm, the cost on recharging and some other problems (such as the slow recharging speed, the low recharging efficiency, etc.) are fully considered. Only the cluster headers and the candidate cluster headers can be recharged, which keeps their residual energy at the same level as that of the common nodes. Therefore, we do not compare the proposed method with other wireless recharging methods. 

**Figure 13:**
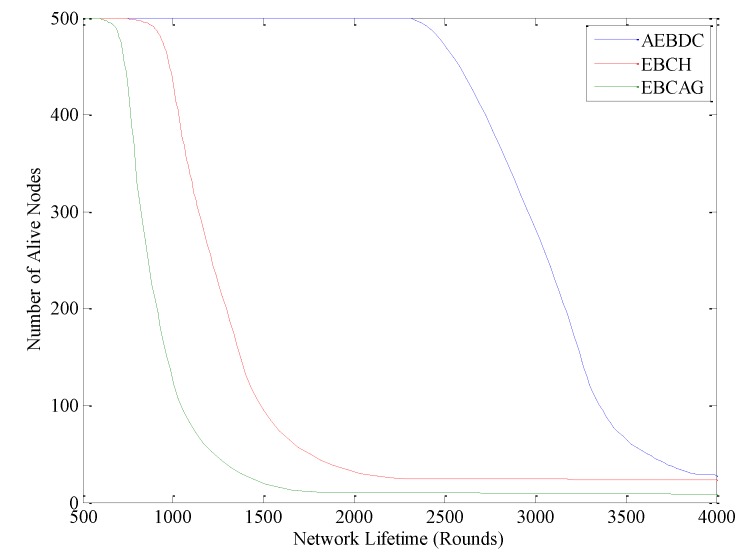
Number of alive nodes in the three algorithms.

Figure 13 shows the comparison of the number of alive nodes among EBCH, EBCAG and AEBDC. It is easy to see that, the performance of EBCAG is the worst. The dead nodes begin to appear after about 800 rounds, and a large number of nodes die after about 1500 rounds. However, in EBCH, it is not until at around 1100 rounds that the dead nodes appear, and the network lifetime ends at around 2000 rounds, which shows better performance than EBCAG. With the help of the non-uniform deployment of nodes and the optimal load distribution strategy for the cluster headers, an approximate balance of energy consumption among CHs in the same annulus is realized in EBCH, so the network lifetime of this data collection method is prolonged to a certain extent. However, in EBCH, once the CHs die, other nodes will also die quickly, and thus the network lifetime will rapidly decrease. While in AEBDC, the network is divided into different sizes of annulus-sectors. Then, according to the amount of data that nodes need to send and receive in each annulus-sector, we calculate out the size of each RCCH to balance the network load. At the same time, the collaboration based multiple-hop data forwarding strategy is adopted in each annulus-sector to further reduce the energy consumption of the CHs. With the running of the network, the energy consumption rate of the CHs becomes lower and lower in AEBDC so that the number of alive nods of it decreases slower than that of EBCH and EBCAG, as shown in Figure 13. That is to say, the energy balance performance of AEBDC is better than other methods.

**Figure 14:**
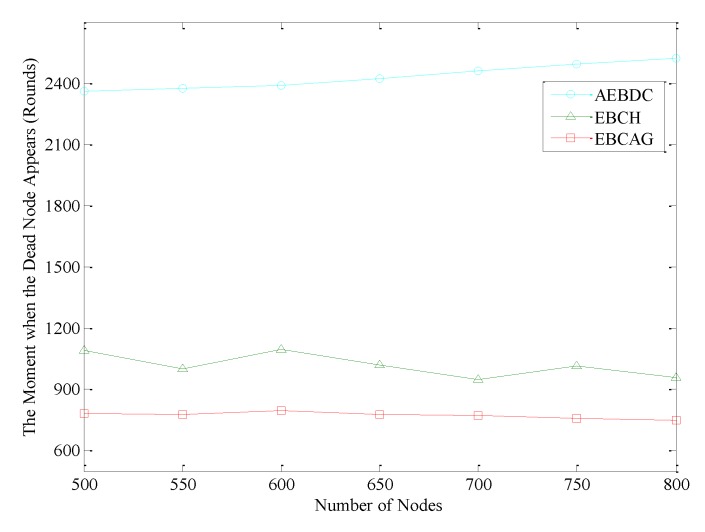
The time when the first dead node appears in the three algorithms.

The time when the first node dies is shown in Figure 14. It is easy to see that in EBCAG, no matter how many nodes are deployed in the network, the moment when the first dead node appears is almost unchanged (at about the 800-th round). The reason is that the optimal radius of the cluster is calculated which minimizes the energy consumption of the whole network. When the total number of nodes increases, the length of radius of each cluster decreases accordingly to maintain the number of nodes within the cluster unchanged essentially. Therefore, the energy consumption of the CHs in EBCAG does not change too much. For EBCH, when the first node dies, the number of data gathering cycles fluctuates to a certain extent with the increase of the number of nodes, and it appears to decrease slightly as a whole. The reason for this tendency is that the number of nodes in each cluster will increase when there are more and more nodes in the network, which enhances the workload of CH. Thus, as the energy consumption of CH increases, the moment when the first dead node appears is ahead of time. That is to say, EBCH is not suitable for the densely deployed network. In AEBDC, although the network load becomes heavier and heavier with the increase of the total number of nodes, the number of nodes in the RCCH increases accordingly. This ensures that more nodes can participate in competing for becoming the cluster header. This effectively balances the energy consumption of each RCCH. Therefore, in AEBDC, the time when the first dead node appears is postponed as the number of nodes increases. In other words, the data collection method proposed in this paper can be applied to various types of networks.

**Figure 15:**
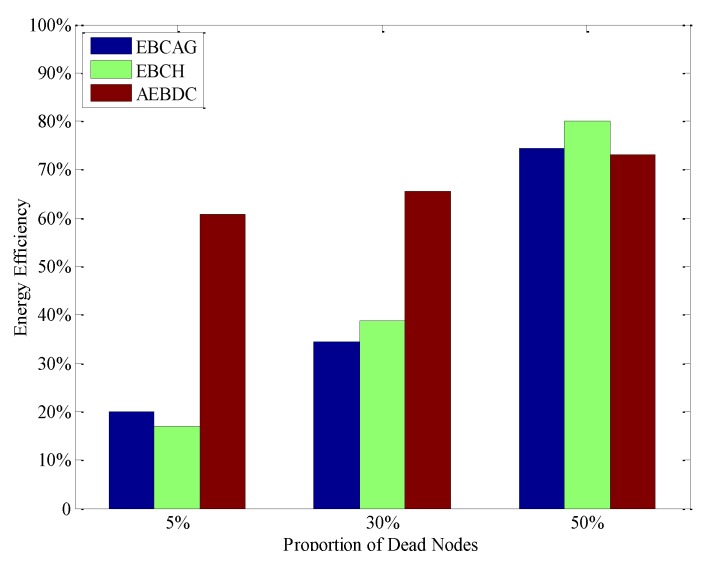
The energy efficiency of the three algorithms.

It is worth mentioning that, in AEBDC, there are a few WCVs in the network to periodically recharge the nodes in each RCCH, and the actual available energy of nodes in AEBDC is greater than that in EBCAG and EBCH. Thus, the “energy efficiency” of the three methods are further analyzed, as shown in Figure 15.

Here, the “energy efficiency” is defined as the ratio between all nodes’ energy consumption and the sum of their initial energy as well as the energy supplied for them. From the simulation result, it is known that the energy efficiency of AEBDC is much higher than that of EBCH and EBCAG when 5% or 30% of nodes die. Although the difference in energy consumption between the CNs and the CHs is reduced due to the cluster-header rotation strategy of EBCAG, it still consumes a lot of energy on calculating the optimal cluster radius as well as reclustering. On the other hand, the EBCH method adopts the local CH rotation scheme as well as the non-uniform deployment strategy to balance the energy consumption of CHs in each annulus. It reduces the expense on CH rotation, but there is still a large difference in energy consumption between cluster heads and non-cluster heads. Thus, the energy efficiency of EBCH is also lower than that of AEBDC. Moreover, the energy consumption of CCHs is approximately balanced in each round of data gathering time in our method, and the recharging scheme also ensures that the CCHs keep working before some common nodes die, which makes full use of energy.

When 50% of nodes die, the energy efficiency of AEBDC is a little lower than the other two algorithms. This is because in AEBDC most of these dead nodes are common nodes and they are located in the outer annuluses. In this case, the energy consumption of CHs in the inner annuluses for forwarding data is great decreased. On the contrary, in EBCH and EBCAG, most of the CHs near the sink exhaust their energy in advance of the common nodes. Thus, the work load on the alive CHs becomes heavier, which increases its energy efficiency to a certain extent.

## 7. Conclusions

In this paper, we have proposed a multi-hop data forwarding strategy on annulus-sectors and a recharging scheme, which balances the energy consumption of sensor nodes in WSNs. Simulation results show that the proposed data collection methods have better performance in prolonging network lifetime and enhancing energy efficiency compared with other strategies. In the future, we will optimize the recharging efficiency of the outer annuli by analyzing the optimal number of WCVs. We will also extend our work to networks where nodes are randomly deployed. 

## Figures and Tables

**Table 1:** Definitions of parameters used in the network.

Symbol	Definition	Unit
*R*	Network Radius	m
*k*	Number of Annuluses	-
*n*	Number of Sectors	-
*N*	Total Number of Nodes	-
*ρ*	Density of Nodes	1/m^2^
*r_t_*	Communication Radius of Node	m
*C*	Battery Capacity of Node	J

**Table 2:** Definition of data collection parameters.

Parameter	Definition	Unit
*r_i_*	Radius of the RCCH in the *i*-th Annulus	m
*W*	Weight of the CCH	-
*l*	The Amount of Data Collected by one Node during a Round of Data Gathering Time	bit
*N_i_*	Average Number of Nodes in Each Annulus-sector of the *i*-th Annulus	-
*E_CH_^i^*	Energy Consumption of One CH in the *i*-th Annulus during a Round of Data Collection	J
*E_elec_*	Energy Consumption of the Sending and Receiving Circuit	nJ × b^−^^1^
*ε_fs_*	Energy Consumption of the Amplifier in Free-Space Model	pJ × (b/m^2^)^−^^1^
*ε_amp_*	Energy Consumption of the Amplifier in Multi-Path Fading Transmission	pj × (b/m^4^)^−^^1^
*d_0_*	The Threshold Distance between the Sender and the Receiver	m
*E_ij_*	The Total Energy Consumption of the Non Cluster Headers in One Annulus-Sector	J
*e*_0_	The Initial Energy of One Node	J
*e_r_*	The Residual Energy of One Node	J
*d_tocenter_*	The Euclidean Distance between the CCH and the Center of the RCCH	m
*α*	Adjustable Parameter	-
*β*	Adjustable Parameter	-
*δ*	Adjustable Coefficient	-
*x*	The Euclidean Distance from any One Node in this Annulus-sector to BS	m
*d_s_*	The Euclidean Distance from any One Node in this Annulus-sector to the Center of the RCCH	m
*θ*	The Angle Deviated from the Center of the RCCH to any One Node	-
*e_t_*	Energy Consumption on Sending One Bit of Data	J
*e_r_*	Energy Consumption on Receiving One Bit of Data	J
*C*’	Buffer Size of Node	bit
*c_i_*	The CH in the *i*-th Annulus	-

**Table 3:** Definitions of wireless recharging parameters.

Symbol	Definition	Unit
*T_i_^r^*	A Round of Recharging Time of WCV	s
*t_ij_^c^*	Time Duration for the WCV to Recharge all Nodes in the *j*-th RCCH of the *i*-th Annulus	s
*t_ij_^m^*	Time Duration for the WCV to Move from the *j*-th RCCH to the (*j +* 1)-th RCCH	s
*l_i_*	The Moving Path Length of the WCV in the *i*-th Annulus during a Round of Recharging	m
*B*	The Initial Energy of WCV at the beginning of each Recharging	J
*R_i_*_,*j*_^(*a*)^	The Total Energy being Recharged to all those Nodes in the *j*-th RCCH of the *i*-th Annulus during the *a*-th Round of Recharging	J
*P_m_*	Energy Consumption of the WCV on Travelling one Meter	J
$E_{i,j}^{r\left(a\right)}$	Total Residual Energy of all those Nodes in the *j*-th RCCH of the *i*-th Annulus at the Moment when the WCV Arrives at this RCCH the *a*-th Time	J
$E_{i,j}^{r(a)}\left(k\right)$	Total Residual Energy of all those CNs in one Annulus-sector of the *k*-th Annulus at the Moment when the WCV Arrives at the *j*-th RCCH of the *i*-th Annulus the *a*-th Time	J
*E_CN_^k^*	Total Energy Consumption of all those CNs in an Annulus-sector of the *k*-th Annulus in *t_r_*	J
*t_r_*	A Round of Data Gathering Time	s
*T_w_^i^*(*a*, *a* + 1)	The Time Period for the Rechargeable Node from its *a*-th Recharging to its (*a +* 1)-th Recharging	s
*v_WCV_*	The Moving Speed of WCV	m/s
*P_c_*	The Recharging Rate of WCV	J/s

**Table 4:** Parameter values.

Parameter	Symbol	Values
Initial Energy of Each Node	*e*_0_	4 J
Maximal Battery Capacity of Each Node	*C*	4 J
Length of Network Radius	*R*	240 m
The Amount of Data Collected by a Node during a Round of Data Gathering Time	*l*	1800 bits
Energy Consumption of the Sending and Receiving Circuit	*E_elec_*	50 Nj/bit
Energy Consumption of the Amplifier in Free-Space Model	*ε_fs_*	10 Pj (b/m^2^)^−1^
Adjustable parameter	*α*	0.5
Adjustable parameter	*β*	0.5
Adjustment coefficient	*δ*	40
The Initial Energy of WCV at the beginning of Each Recharging	*B*	100 J
Energy Consumption of the WCV on Travelling One Meter	*P_m_*	0.0023 J/m

**Table 5:** The average number of nodes in each RCCH and their total energy consumption in a round of data collection.

		*k* = 3, *n* = 8	*k* = 4, *n* = 5	*k* = 5, *n* = 4
The Average Number of Nodes in the RCCH	of the First Annulus	3	3	3
	of the Second Annulus	2	3	3
	of the Third Annulus	2	3	3
	of the Fourth Annulus	-	2	2
	of the Fifth Annulus	-	-	1
Total Energy Consumption of Nodes		0.4174	0.5529	0.6445

**Table 6:** Average energy consumption of nodes in the RCCH under different values of *k*.

		*k* = 3, *n* = 8	*k* = 4, *n* = 5	*k* = 5, *n* = 4
Average Energy Consumption of Nodes in the RCCH	of the First Annulus	0.0037	0.0040	0.0051
	of the Second Annulus	0.0045	0.0040	0.0049
	of the Third Annulus	0.0030	0.0037	0.0047
	of the Fourth Annulus	-	0.0035	0.0049
	of the Fifth Annulus	-	-	0.0050

**Table 7:** Total recharging efficiency and energy distribution of WCV.

	*k* = 3, *n* = 8	*k* = 4, *n* = 5	*k* = 5, *n* = 4
Total Recharging Efficiency of WCV	70.19%	59.9%	57.67%
Percentage of Energy Consumption on Moving	17.34%	21.95%	32.21%
Percentage of the Residual Energy after Returning Back to BS	12.47%	18.15%	10.12%
